# The Community Coevolution Model with Application to the Study of Evolutionary Relationships between Genes Based on Phylogenetic Profiles

**DOI:** 10.1093/sysbio/syac052

**Published:** 2022-07-29

**Authors:** Chaoyue Liu, Toby Kenney, Robert G Beiko, Hong Gu

**Affiliations:** Department of Mathematics and Statistics, Dalhousie University, Halifax, NS B3H 4R2, Canada; Faculty of Computer Science, Dalhousie University, Halifax, NS B3H 4R2, Canada; Department of Mathematics and Statistics, Dalhousie University, Halifax, NS B3H 4R2, Canada; Faculty of Computer Science, Dalhousie University, Halifax, NS B3H 4R2, Canada; Department of Mathematics and Statistics, Dalhousie University, Halifax, NS B3H 4R2, Canada

## Abstract

Organismal traits can evolve in a coordinated way, with correlated patterns of gains and losses reflecting important evolutionary associations. Discovering these associations can reveal important information about the functional and ecological linkages among traits. Phylogenetic profiles treat individual genes as traits distributed across sets of genomes and can provide a fine-grained view of the genetic underpinnings of evolutionary processes in a set of genomes. Phylogenetic profiling has been used to identify genes that are functionally linked and to identify common patterns of lateral gene transfer in microorganisms. However, comparative analysis of phylogenetic profiles and other trait distributions should take into account the phylogenetic relationships among the organisms under consideration. Here, we propose the Community Coevolution Model (CCM), a new coevolutionary model to analyze the evolutionary associations among traits, with a focus on phylogenetic profiles. In the CCM, traits are considered to evolve as a community with interactions, and the transition rate for each trait depends on the current states of other traits. Surpassing other comparative methods for pairwise trait analysis, CCM has the additional advantage of being able to examine multiple traits as a community to reveal more dependency relationships. We also develop a simulation procedure to generate phylogenetic profiles with correlated evolutionary patterns that can be used as benchmark data for evaluation purposes. A simulation study demonstrates that CCM is more accurate than other methods including the Jaccard Index and three tree-aware methods. The parameterization of CCM makes the interpretation of the relations between genes more direct, which leads to Darwin’s scenario being identified easily based on the estimated parameters. We show that CCM is more efficient and fits real data better than other methods resulting in higher likelihood scores with fewer parameters. An examination of 3786 phylogenetic profiles across a set of 659 bacterial genomes highlights linkages between genes with common functions, including many patterns that would not have been identified under a nonphylogenetic model of common distribution. We also applied the CCM to 44 proteins in the well-studied Mitochondrial Respiratory Complex I and recovered associations that mapped well onto the structural associations that exist in the complex. [Coevolution; evolutionary rates; gene network; graphical models; phylogenetic profiles; phylogeny.]

Comparative studies can provide useful insights into selection and adaptation of organismal traits in concert with their evolutionary history (Sanford et al. 2002). The types of traits that can be assessed in this framework are broad and can include morphology, behavior, physiology, and ecology (Peiman and Robinson 2017). For example, a study of traits in plants showed positive correlations between woodiness and tannin frequency, and a negative correlation between tannin frequency and alkaloid frequency, which they hypothesize is related to chemical defense (Silvertown and Dodd 1996). Many examples in animals have been observed as well, such as the association between coloration and behavior in snakes (Brodie III 1992). Comparative-genomic techniques can be used to identify homologous genes that underpin a multitude of traits (Koonin et al. 2000; Haubold and Wiehe 2004). Genes can exhibit similar patterns of presence and absence (Pellegrini et al. 1999) across a set of genomes for reasons such as participation in a common biochemical pathway, physical linkage, or colocalization on a mobile genetic element such as a plasmid (Fraser et al. 2004; Bowers et al. 2004; Cong et al. 2019). Examination of these patterns can reveal important information about related functions (e.g., participation in a common biochemical pathway) and common pathways of lateral gene transfer (LGT). A well-established approach to represent presence and absence patterns among genes is the construction of *phylogenetic profiles*, binary vectors that summarize the presence and absence of genes across a set of genomes, effectively treating each gene as a separate trait (Pellegrini 2012; Niu et al. 2017; Moi et al. 2020).

The success of phylogenetic profiling depends on the use of appropriate measures to express the distance and similarity between profiles. Approaches include the Hamming distance (Pellegrini et al. 1999), mutual information (Huynen et al. 2000), Pearson correlation (Sadreyev et al. 2015), and the hypergeometric test (Wu et al. 2003). Although effective, these approaches do not take phylogenetic effects into account. Since closely related genomes are more likely to share similar gene content, they are likely to have an outsized influence on profile comparisons relative to their phylogenetic diversity. Thus, the genomes connected by the phylogenetic tree are not independent (Pyron et al. 2015), which will violate the assumptions of these methods, and therefore skew results. Large genomic data sets are often imbalanced due to high relative abundance or oversampling of certain genomes; for example, the over-representation of pathogen isolates in the set of complete prokaryotic genome sequences (Alcaraz et al. 2010).

Several heuristic approaches have been developed to account for phylogenetic effects in profiles. For example, Jothi et al. (2007) and Sadreyev et al. (2015) used a null distribution of the similarity scores inferred by sampling the genomes to estimate the impacts of phylogenetic correlation; while von Mering et al. (2003) corrected for biases in the number of sequenced genomes by collapsing the genomes within the same clade into one single node if a specific gene pair has the same phyletic pattern in these genomes. Cokus et al. (2007) first ordered the genomes within profiles and enumerated runs of consecutive matches so that the co-occurrences concentrated in part of the tree would be considered as only one run. The shared underlying idea of these methods is the application of a weighting scheme to the genomes in order to counteract phylogenetic effects. These methods can be computationally efficient and feasible for large-scale analysis (Szklarczyk et al. 2021; Tremblay et al. 2021), but they are *ad hoc* approaches that do not properly model the underlying evolutionary processes.

In contrast with weighting approaches, evolutionary models aim to explain the distribution of genes by modeling the correlation patterns of gain and loss on a phylogenetic tree. Model-based approaches include CoPAP which uses a stochastic mapping approach to detect coevolving gene families (Cohen et al. 2012, 2013), the CLustering by Inferred Models of Evolution (CLIME) algorithm that was developed to model gene evolution in eukaryotes (Li et al. 2014), and the Count software package for the analysis of numerical profiles using phylogenetic birth-and-death models (Csűös 2010). However, Count was not specifically developed for binary traits; CLIME assumes that each gene has a single gain event in evolution which is not suitable for prokaryotes which have high rates of gene transfers (Vos et al. 2015); and CoPAP assumes that the gain rate and loss rate independently vary among genes rather than explicitly modeling the interactions during evolution. Pagel (1994) developed a likelihood-based coevolutionary method that specifically tests the evolutionary correlations between pairs of binary traits. In Pagel’s method, each pair of binary traits is evaluated under both independent and dependent models, and a likelihood ratio test is applied to infer whether there is significant evidence suggesting two traits evolved dependently. Although Pagel’s correlation model performed well in previous studies of detecting functionally linked genes (Barker and Pagel 2005; Liu et al. 2018), Pagel’s method is computationally expensive and it cannot directly infer the direction (positive/negative) of the correlation. In addition, there is a more general concern regarding the phylogenetic comparative methods represented by Pagel’s correlation model raised by Maddison and FitzJohn (2015) and Uyeda et al. (2018) that comparative methods may overestimate the evidence for the correlation of the patterns caused by singular events which they refer to as Darwin’s scenario. Darwin’s scenario occurs when two traits have a single origin on the same lineage and are then inherited by nearly all species in the descendant clade, resulting in (almost) perfectly codistribution. This “within-clade pseudoreplication” could lead to dubious conclusions such as a significant association between fur and middle earbone (Maddison and FitzJohn 2015).

Furthermore, most of the existing methods such as Pagel’s correlation method and distance-based methods can only be applied to pairs of genes. However, studying phylogenetic profiles in higher-order groups (such as triplets and quadruplets) can offer a more-sensitive approach to detecting complex patterns of correlation (Wu et al. 2005). Direct-coupling analysis is a class of methods often used to infer direct relationships between residues in biological sequences that can deal with conditional dependency by taking the inverse of the covariance matrix, but it is mainly for continuous data and phylogeny naive so it is highly dependent on the sampling of the genomes (Morcos et al. 2011; Baldassi et al. 2014).

Here, we propose the Community Coevolution Model (CCM), a new method that directly infers the strength and direction of the interactions among genes during the evolutionary process. For a pair of genes, CCM is more efficient in that it fits only one model instead of three (one separate model for each gene and one dependent model) in Pagel’s method and is approximately five times faster than Pagel’s method when tested on phylogenetic trees with 500 tips (performed on a server running Linux with a 2.67 GHz CPU and 18 GB RAM). Although maximum-likelihood estimation (MLE) is still a time-consuming procedure compared to other heuristic methods based on standard metrics, our method provides more biological insights such as the evolutionary rates, significance levels and directions (positive/negative) of interactions, and more importantly our method can be extended to model multiple genes as a community to discover more-complex associations. We also develop a simulation procedure to generate phylogenetic profiles with adjustable extents of evolutionary interactions that can be used as benchmark data for evaluating comparative methods.

## Materials and Methods

### The Community Coevolution Model

In our CCM, we consider whether sets of two or more genes have potential associations on a given phylogenetic tree, in particular whether the transition (gain or loss) of any gene within the community is affected by the current states of other members. Associations between genes can be positive if genes tend to be gained and lost together, and negative if the gain of some genes in a set appears to be associated with the loss of others in the same set. Gene sets that show evidence of associations are termed as a “community” in our model.

We formulate the transition rate $\tau$ for one gene as a function of its intrinsic rate of gain and loss $\mu$, and the association factor $\omega$ depending on the current states of all other genes in the community,


(1) \begin{equation*}\tau = \mu \times \omega.\end{equation*}

To further specify our model, we use the following notation:

$n$ is the total number of genes in the community;

$\mathcal{S} = \{S_1, S_2, ..., S_{2^n}\}$ is the state space of a community consisting of $n$ genes;

$S_i = \{ x_{i,k} ; k = 1,...,n \}$ is a vector of size $n$. $x_{i,k}$ denotes the state of the specific $k$th gene when the community state is at $S_i$. We define $x_{i,k} = -1$ when the $k$th gene is absent and 1 when the $k$th gene is present;

$\mathcal{B} = \{ \beta_{hk}; k, h = 1, ..., n\}$ is a symmetric $n \times n$ matrix whose off-diagonal entries are the coefficients of interaction between every pair of genes and diagonal entries indicate half the difference between the gain and loss rates of each gene;

$\mathcal{A} = \{\alpha_1, \alpha_2, ... , \alpha_n\}$ is a vector of size $n$ containing the intrinsic rate which is the mean of gain and loss rates for each gene;

The instantaneous transition rate for a specific gene $k$ when the whole gene community is in state $S_i$ is defined in the log scale as


(2) \begin{equation*}\label{log_scale}\log(\tau_{i,k}) = \alpha_k - \beta_{kk} x_{i,k} - \sum_{h \neq k}^n \beta_{hk} x_{i,k} x_{i,h}.\end{equation*}

Positive $\beta_{hk}$ means the $k$th and $h$th genes are positively associated, thus if the current states of $k$th and $h$th genes are the same ($x_{i,k} x_{i,h}=1$), the last term tends to reduce the rate of change for gene $k$; and vice versa for negative values of $\beta_{hk}$.

By taking the exponential of equation (2), we have the final model as


(3) \begin{equation*}\label{eq:cal_rate}\tau_{i,k} = \underbrace{exp(\alpha_k - \beta_{kk} x_{i,k})}_{\mu} \cdot \underbrace{exp \left( - \sum_{h \neq k}^n \beta_{hk} x_{i,k} x_{i,h} \right)}_{\omega},\end{equation*}

where the first part represents the intrinsic gain/loss rates of gene $k$ and the latter part represents the influence from the community.

We model the gene state changes along a phylogenetic tree as a continuous-time Markov process and assume the instantaneous rate for all transitions involving more than one gene is 0. The transition rate matrix $Q = \{q_{ij}; i, j =1, ..., 2^n\}$ , where element $q_{ij}$ denotes the rate of the community departing from state $S_i$ and arriving in state $S_j$, can be constructed in accordance with the following rule,


(4) \begin{equation*}q_{ij} = \left\{\begin{matrix}\tau_{i,k}, & i \neq j \text{ and } S_i - S_j = \pm 2\mathbf{e}_k\\-\sum_{i' \neq i}{q_{ii'}}, & i = j \\0, & \text{otherwise}\end{matrix}\right.,\end{equation*}

where $\{\mathbf{e}_k; k = 1, ..., n\}$ denote the standard basis vectors of all 0’s except the $k$th element as 1 so that $S_i - S_j = \pm 2\mathbf{e_k}$ indicates that only the $k$th gene changes state.

### Constructing the likelihood function given the tree

**Figure 1.**
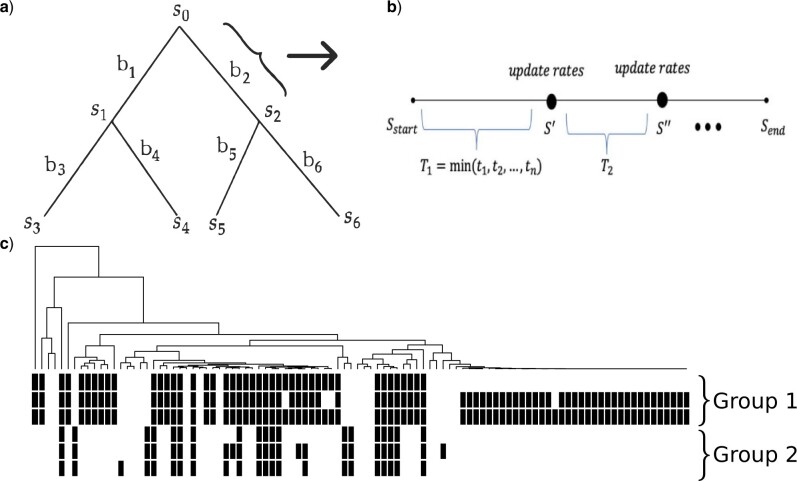
a) A phylogenetic tree with four tips: $s_i (i = 0, ..., 6)$ represents the state at each node and $b_i (i = 1, ..., 6)$ denotes the branch length. b) An illustration of the simulation process on one branch. $S$ denotes the community states and $T$ indicates the time that there is a transition out of current state. The process ends when the total transition time is beyond the branch length. c) A random realization of two groups of correlated profiles of Size 3 generated by our simulation procedure. The interaction coefficient is set to be 1.5 within a group and 0 between groups. Each row is a profile and each black bar denotes presence of the gene.

In addition to the Markov assumption, we also assume that transitions on separate branches are independent. This means that the distribution of the state at the end of a given branch depends only on the starting state of that branch. The computational cost of constructing the likelihood function could be expensive as we need to sum over all the possible combinations of the states at each internal node, but it can be reduced by applying Felsenstein’s pruning algorithm (Felsenstein 1973). The pruning algorithm is a dynamic-programming approach that takes advantage of the nested structure of the tree and computes the likelihood for the given tree recursively. By applying the pruning algorithm, the likelihood function $L$ of the tree in Figure 1a can be formatted as


(5) \begin{equation*}\label{eq:like0}\begin{aligned}L(\Theta; \mathcal{T}, \mathbb{X} ) = \sum_{s_0} & \left[ \left(\sum_{s_1} P_{s_0,s_1}(b_1) P_{s_1,s_3}(b_3) P_{s_1, s_4}(b_4) \right) \right. \\& \left. \times \left(\sum_{s_2} P_{s_0,s_2}(b_2) P_{s_2, s_5}(b_5) P_{s_2, s_6}(b_6)\right)\right].\end{aligned}\end{equation*}

In this way, the likelihoods for subtrees can be reused and the computational complexity is reduced to linear in the number of leaves in the tree. Then, the negative log-likelihood function $-\log(L(\Theta; \mathcal{T}, \mathbb{X} ))$ is minimized to acquire the maximum likelihood (ML) estimates of the parameters by using a quasi-Newton optimizer (*nlminb* in *R*, version 4.0.2) (Paradis et al. 2004).

### Inference and Regularization of the ML Estimates

Due to the complexity of the likelihood function, it is necessary to assess whether the optimizer is going to provide acceptable estimates. We examined two ways to obtain the standard error of estimates: first, the parametric bootstrap method that simulates a large number of profiles using the estimated parameters and calculates the standard deviation of the estimates using the bootstrap samples; second, the analytical approach based on likelihood theory which utilizes the numerically approximated Hessian matrix $H$ of the objective function $-\log(L(\Theta))$, with the standard error given as $\textrm{se} = \sqrt{\textrm{diag}(H^{-1})}$. Using this estimated standard error, a Z-test is conducted to obtain the *P*-value for the hypothesis $\beta_{hk}=0$. The bootstrap method is obviously more time-consuming, but we can use the bootstrap to assess the accuracy of the likelihood asymptotic for finite samples. The performance of these two methods is compared in the Results section.

As tree and community size increases, the likelihood function can become extremely complicated. A potential problem with the MLE procedure is overshooting, which happens when the parameter estimators diverge substantially from the true values due to a flat likelihood surface. To avoid the overshooting problem, we apply $l_2$-regularization on the parameters and have the penalized objective function


(6) \begin{equation*}-\log\left(L(\Theta; \mathcal{T}, \mathbb{X} )\right) + \lambda \cdot \left(||\Theta||_2^2\right)\!,\end{equation*}

where $\lambda$ is the tuning parameter. Unlike setting a boundary for parameters or allowing a large error tolerance, which could cause an early stop of the optimization process to avoid overshooting, the regularization approach leads to more stable estimations by adding smoothness to the surface of the likelihood function.

The $l_2$-regularization is not meant for model sparsity, but only for dealing with computational issues of likelihood singularity in some occasional cases, and therefore it is not needed in most analyses which avoids unnecessary bias. When it is needed, a reasonable $\lambda$ is desired to avoid the overshooting problem but without introducing too much bias into the estimation. The condition number, which is the ratio of the largest eigenvalue to the smallest of the Hessian matrix, describes the rate of convergence of the optimization (Thacker 1989), and can be used as a guide to find a proper $\lambda$. To provide a rule of thumb, we find that a condition number below $200$ generally indicates a successful convergence without the overshooting problem.

### Simulation Procedures

To simulate the coevolutionary relationships among genes, we use the framework of CCM that the transition rates of one gene depend on the states of other genes in the community. The procedure for simulating the evolution of a gene community of size $n$ on one branch can be summarized as below:

Input: the starting state of the community $\mathcal{S}$, a user-defined coefficient matrix $\mathcal{B}_{n\times n}$, user-defined intrinsic rates $\mathcal{A}_0$ and branch length $b$.
  1. Substitute the current states and user-defined parameters into Formula 3 to calculate the current transition rate for each gene $\tau_{h}, h=1, ..., n$.
  2. Sample the transition time for each gene from the exponential distribution, $t_h \sim Exp(\tau_{h}), h = 1, ..., n$.
  3. Find the gene $k$ with the minimum transition time, $T_{\min} = \min\{t_1, t_2, ... , t_n\}$.
  4. If $T_{min} \leq b$, update the state of gene $k$ in $\mathcal{S}$ with the opposite state and if $T_{\min} > b$, do not update the gene state. Then update the branch length $b = b - T_{\min}$.
  5. Repeat steps 1–4 until $b \leq 0$.
Output: the new state of the community $\mathcal{S}'$.

An illustration of the evolutionary process on one branch is shown in Figure 1b. Then, the end state $\mathcal{S}_{\text{end}}$ will be the starting state for the next adjacent branches. The same procedure will be applied to all branches sequentially from the root to the tips. Figure 1c shows a simulation example of six genes in two groups of Size 3 using the interaction matrix which has within-group interaction coefficients equal to 1.5, indicating strong relationships and between-group interaction coefficients equal to 0, indicating independent evolution.

### Analysis of Genomes from Class Clostridia

We applied our method to the draft assembly of the bacterium “*Lachnospiraceae* bacterium 3-1-57FAA-CT1” (abbreviated as LZ), which was isolated from a biopsy retrieved from the transverse colon of a female Crohn’s Disease patient at the time of colonoscopy (Liu et al. 2018). LZ is of interest because its genome size is very large compared to most of its immediate neighbors (6505 protein-coding genes as compared with a median of 3124 in our complete data set of Clostridia) and identifying sets of genes with shared patterns of gain and loss may yield insights into its ecological role in the host. 658 completed and draft genomes from class Clostridia were retrieved from the National Center for Biotechnology Information (NCBI) for the comparative analysis of LZ. The phylogenetic tree was built through the AMPHORA2 pipeline (Wu and Scott 2012) and RAxML-HPC (Stamatakis 2006) using their concatenated, conserved protein sequences and another set of eight outgroup genomes from class Bacilli and phyla Actinobacteria and Proteobacteria were used for rooting. The phylogenetic profiles were constructed by comparing the complete set of LZ (6505 predicted genes) against all other genomes using rapsearch (Ye et al. 2011). Before our analysis, we firstly filtered out the genes that are very rare (present in $< 1\%$ genomes) or very common (present in $>99\%$ genomes) and obtained the final data set of 3786 profiles. The Markov Clustering algorithm (MCL) was also used to obtain clusters of genes. MCL is a graph-based clustering method that simulates random walks within the graph to reflect the cluster structure based on the idea that random walks are more likely to stay in one natural cluster than to move across clusters (van Dongen and Abreu-Goodger 2012).

## Results

### Results on Simulated Data

#### Evaluation of model estimates

**Figure 2.**
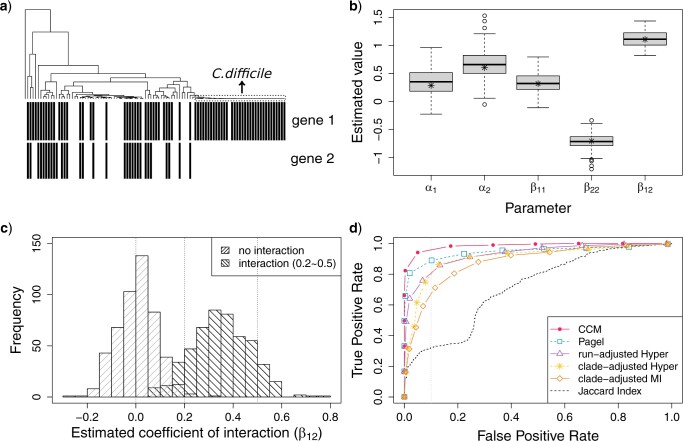
Estimation of the parameters using simulated pairs: a) Two phylogenetic profiles from the real data set; b) Estimated parameter values from CCM based on 100 simulated pairs using the parameters estimated from the two profiles in (a). The “*” represents the true parameters used in simulation. Evaluation of the interaction in pairs: c) The distributions of the estimated coefficients of interaction of the “no interaction” group and the “with interaction” group; d) the ROC curves of detecting the significant linkages by Jaccard Index, Pagel’s correlation method, clade-adjusted mutual information and hypergeometric, run-adjusted hypergeometric and our CCM model.

We first evaluated the performance of CCM on simulated data. We used the parameters estimated from one pair of flagellar genes in the real data set, with profiles shown in Figure 2a. We simulated 100 pairs of genes using the parameters estimated from these two genes. The results are shown in Figure 2b. We see that the estimates are distributed around the true parameter values. Furthermore, we compared the estimates of the standard error based on the Hessian matrix and the parametric bootstrap and Table 1 shows that the estimation results of two methods are consistent.

**Table 1:** Comparison of estimated standard error using the parametric bootstrap and analytical Hessian methods based on the simulations in Figure 2

	$\alpha_1$	$\alpha_2$	$\beta_1$	$\beta_2$	$\beta_{12}$
Bootstrap SE	0.282	0.215	0.107	0.099	0.090
Hessian SE	0.382	0.255	0.104	0.105	0.085

#### Detection of significant interactions between genes

We next used our simulation approach to examine the ability of the CCM to distinguish genes with associations from those that do not interact. To evaluate the performance of CCM, we simulated 500 gene pairs with no interaction ($\beta_{12} = 0$) as negatives and 500 gene pairs with interactions ($ \beta_{12}$ uniformly drawn between 0.2 and 0.5) as positives. Figure 2c shows the distributions of the estimated coefficients of interaction in two groups: the mean value for the “no interaction” group is 0.0046 ($\pm$ 0.0807) and for the “interaction” group is 0.3497 ($\pm$ 0.1173). We also compared the performance of Pagel’s correlation test method, the Jaccard Index ($ = \frac{\text{number of genomes that have both genes}}{\text{number of genomes that have either of genes}}$) and two heuristic methods: hypergeometric with consecutive runs method (Cokus et al. 2007) and mutual information with clade adjustment method (von Mering et al. 2003). We evaluated both clade-adjusted and runs-adjusted methods with four different metrics (Hamming, Jaccard Index, Hypergeometric test, and Mutual Information) using simulated data, and we found that the hypergeometric test works best. Thus, we also included the clade-adjusted Hypergeometric test for comparison since it performed better than the method proposed in their original paper (clade-adjusted mutual information). From the ROC curve shown in Figure 2d, our CCM method obtained the highest AUC score of 0.9521 followed by Pagel’s correlation model (0.8968), run-adjusted Hypergeometric (0.838), clade-adjusted Hypergeometric (0.7665), and clade-adjusted Mutual Information (0.7215). The Jaccard Index, being a nonphylogenetic method had the lowest AUC score (0.609).

#### Identifying Darwin’s scenario of codistribution

**Figure 3.**
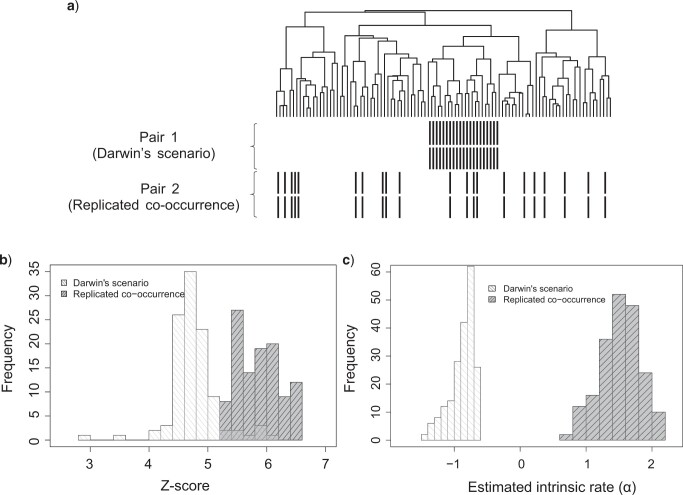
Comparison between Darwin’s scenario and replicated co-occurrence: a) An example of Darwin’s scenario (Pair 1) and replicated co-occurrence (Pair 2); b) The distributions of the Z-scores ($\frac{\beta_{12}}{se(\beta_{12})}$) for the two scenarios; c) The distributions of the estimated intrinsic rates for the two scenarios.

Under Darwin’s scenario, there is a single concurrent origin for two genes leading to the perfect codistribution across all species within a clade as in the example shown in Figure 3a. As Darwin’s scenario provides little evidence for coevolution, it is of interest to distinguish such scenarios from a replicated coevolution scenario that has multiple disjoint instances of a given trait. Both scenarios are considered significantly correlated by CCM due to their perfect codistribution, but the replicated coevolution scenario yields stronger significance scores and has much higher intrinsic rates. We demonstrate this difference using 100 simulated data sets. In each simulation, we randomly generate a 100-tip tree, one pair of genes that have co-occurrences concentrated in one random clade chosen uniformly across all clades as Darwin’s scenario and another pair of genes that have same number of co-occurrences spreading across the tree as the replicated codistribution scenario. Although both scenarios produce significant results (*P*-value$ < 0.005$), replicated co-occurrence tends to achieve greater significance scores (Fig. 3b). Very few of the Darwin scenarios would be deemed significant in a real data analysis with correction for multiple testing. Another distinguishing feature between these scenarios is the estimated intrinsic rate $\alpha$, with gene pairs simulated under Darwin’s scenario having much lower estimates of $\alpha$ ($-0.88 \pm 0.18$) than under replicated coevolution scenario ($1.51 \pm 0.29$) as shown in Figure 3c.

#### Modeling multiple genes as a community to reduce pairwise false-positive links

**Figure 4.**
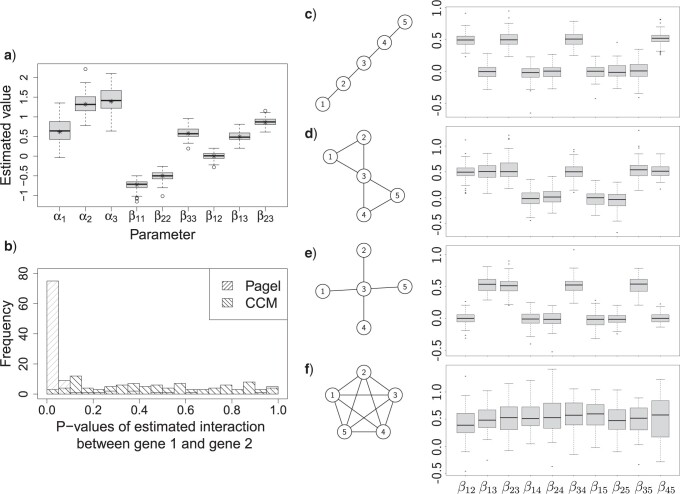
Evaluation of the conditionally independent links in the simulated triplets: a) Estimated parameter values from CCM based on 100 simulated triplets. The sign “*” indicates the true parameters. b) The *P*-values of the conditionally independent pairs (genes $1$ and $2$) inferred by our CCM model and Pagel’s model. Simulation of four association-network structures: c) line, d) partially connected, e) star, and f) fully connected. The networks on the left demonstrate the structures and the box-plots on the right show the estimated coefficients of interactions within the community. All the edges have an interaction coefficient ($\beta_{ij}$) of 0.5.

Most comparative methods (e.g., Pagel’s method and all the methods based on distance or similarity metrics) use pairwise comparisons. By modeling more than two genes as a community, the CCM can screen out false-positive links that can be caused by genes that show pairwise evidence for coevolution but are conditionally independent when other genes are taken into consideration. For example, consider a community of three genes where Gene $3$ is directly related to both $1$ and $2$, but there is no direct connection between Genes $1$ and 2. Pairwise methods will often falsely identify a significant connection between Genes 1 and 2. We simulated 100 triplets of genes with this structure where Gene 3 is moderately linked to Gene 1 ($\beta_{13} = 0.5$) and is strongly linked to Gene 2 ($\beta_{23}$ = 0.8), but Genes 1 and 2 are independent conditional on the presence of Gene 3 ($\beta_{12} = 0$). As shown in Figure 4a, CCM correctly estimates the true parameters. We also ran Pagel’s model over the same simulation data in pairs (three pairs for each group of three genes, so 300 pairwise comparisons in total). From Figure 4b which shows the distribution of estimated *P*-values on the conditionally independent linkage between Genes $1$ and 2, we can see that our method resulted in the desired uniform distribution of *P*-values while Pagel’s method shows $76\%$ of estimates had *P*-value $<0.05$.

#### Recovery of community structures

To evaluate the ability of CCM at recovering the relationships among more genes within a community, we simulated four basic network topologies of a five-node community: i) a line structure; ii) a star structure where the Node 3 acts as the hub; iii) a partially connected network where Node 3 acts as the bridge that connects two subgroups; and iv) a fully connected network. For each structure, we simulated 100 data sets with an interaction coefficient of 0.5 for all links. As shown in Fig. 4c–f, CCM successfully reveals the linkages within the community. Unlike the pairwise methods that will tend to result in a densely connected network due to false positives among the conditionally independent pairs, our community model provides clear insights in finding the importance of the members (e.g., hub genes), and complex dependency structures within the community.

As community size increases, the Q matrix dimension increases quickly which dramatically slows down the evaluation of the log-likelihood values. Thus, our current method can only handle a small number of genes in a community. Table S1 of the Supplementary material available on Dryad at https://doi.org/10.5061/dryad.p8cz8w9rd provides the approximate running time of our program as a reference for different community and tree sizes. For large groups, one strategy is to run all-versus-all pairwise comparisons first to construct a gene-interaction network, which is usually very densely linked at this stage. We then run all the triplets within the network to remove the conditionally independent links. We can continue to examine all the subnetworks of Size 4 or 5 to further prune the network to the desired sparsity.

#### Effect of tuning parameters

We also evaluated the influence of the tuning parameters on the MLEs. We simulated 100 gene pairs with random parameters and expected that the overshooting problem may happen to some of the pairs. These problematic cases will have the estimations of parameters far away from true values, like the outliers in Figure S1 of the Supplementary material available on Dryad. Then, we added the regularization term and increased the tuning parameter $\lambda$ gradually. For each simulated pair, the mean squared error (MSE = $\frac{1}{5}\sum_{i=1}^5(\hat{\theta}_i - \theta_i)^2$) of the estimators (five parameters for two genes) is reported. From Figure S1a of the Supplementary material available on Dryad, we can see that the tuning parameters mainly have a large impact on those outliers. The condition number plot (Fig. S1b of the Supplementary material available on Dryad) shows that when we increase the tuning parameter to make the condition number of the Hessian matrix below 200, the overshooting problems with those outliers were effectively solved.

### Results on Prokaryotic Data

#### Model comparisons

**Figure 5.**
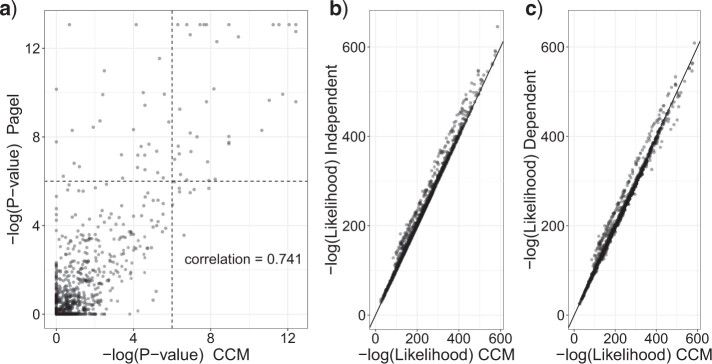
The comparison of significance of pairwise linkages by two methods: the horizontal axis is the $-\log_{10}(P$-value) of CCM and the vertical axis is the $-\log_{10}(P$-value) of Pagel’s approach; the correlation between the *P*-values for the two methods is 0.741. b,c) The comparison of the goodness-of-fit of models to data between the independent model (four parameters), dependent model (eight parameters), and our CCM model (five parameters). The independent model and dependent model are the two components of Pagel’s approach required for the likelihood ratio test.

The computational cost of running a single pairwise profile comparison using Pagel’s model was around 15 s (performed on a server running Linux with a 2.67 GHz CPU and 18 GB Ram). Since the entire data set requires ($\binom{3786}{2} = 7,165,005$) such comparisons, an exhaustive evaluation of Pagel’s method is infeasible. Instead, we focused on a complete pairwise evaluation of 50 adjacent genes to compare against our coevolutionary model. The software we used to implement Pagel’s approach is BayesTraitsV3 (Meade and Pagel 2017). A comparison of the negative logarithm of the *P*-values inferred by the two methods yielded a correlation coefficient of 0.741 as shown in Figure 5a (*P*-values have been adjusted for multiple correlated tests using the Benjamini–Yekutieli (BY) method (Benjamini and Yekutieli 2005)). Applying a log *P*-value threshold of $6$, we found that both methods agreed on the significance or nonsignificance of 1188 (96.98 $\%$) comparisons. Twenty-nine (2.37 $\%$) comparisons gave a significant result with the Pagel test but not with the coevolutionary model, while the opposite result was seen in the remaining 8 (0.65 $\%$) pairwise comparisons. After examining the discordant pairs in the top-left corner of Figure 5a, we found that a common issue for Pagel’s model is that most of these pairs reached the default maximum rate of 100 (the mean branch length of the tree has been scaled to 0.1 as suggested by the authors (Meade and Pagel 2017)), which indicates that Pagel’s dependent model may overestimate the likelihood of correlated evolution because of overshooting and therefore detect more false-positive links. One example of the estimated transition rates by the two methods is compared in Table 2. Pagel’s dependent model has a strange transition rate matrix where the transition rate from (0,0) to (0,1) is abnormally large (98.575) and the transition rate from (1,0) to (0,0) is 0, which may suggest that Pagel’s eight-parameter dependent model may be overparametrized and therefore overestimate the likelihood of dependent evolution.

**Table 2:** The transition rate matrices inferred by Independent and Dependent models of Pagel’s method and the CCM. The gene pair (GI:511537597 and GI:496550319) in this table is considered strongly correlated by Pagel’s method (*P*-value $=$ 0.00011), but not by the CCM (interaction coefficient $=$ 0.0832; *P*-value $=$ 0.283)

	0, 0	0, 1	1, 0	1, 1
(a) Independent model $-\log$(likelihood) $=$ 283.183				
0, 0	–	2.143	0.506	0
0, 1	0.304	–	0	0.506
1, 0	1.866	0	–	2.143
1, 1	0	1.866	0.304	–
(b) Dependent model $-\log$(likelihood) $=$ 271.4941				
0, 0	–	98.575	0.375	0
0, 1	10.190	–	0	0.447
1, 0	0	0	–	2.139
1, 1	0	2.267	0.0003	–
(c) CCM $-\log$(likelihood) $=$ 283.015				
0, 0	–	2.158	0.441	0
0, 1	0.335	–	0	0.521
1, 0	2.188	0	–	2.548
1, 1	0	1.853	0.284	–

We further evaluated the goodness-of-fit of the two methods to the real data by comparing the likelihood scores. As shown in Figure 5b,c, the CCM model obtains a significantly lower negative log-likelihood than Pagel’s independent model (*P*-value $< 2.2 \times 10^{-16}$) and dependent model (*P*-value = 0.00217), which suggests that our model generally has better fit to the real data, even though our CCM model (five parameters) has fewer parameters than Pagel’s dependent model (eight parameters).

#### Gene clustering based on significant pairwise linkages

To discover sets of genes that collectively show evidence of correlated gains and losses, we performed a full pairwise comparison over the genes of LZ using CCM. There are in total 1918 genes annotated with gene ontology (GO) biological process terms, which were used to evaluate the gene functional similarities of the linkages and for the GO enrichment analysis. All GO annotations of genes were retrieved from the UniProt database (UniProt Consortium 2015). We use Wang’s graph-based method (Wang et al. 2007) to measure the semantic similarity of GO terms, which produces a score between 0 and 1 for a given pair of GO terms and higher values represent more functional similarity (Wang et al. 2007; Yu et al. 2010). Figure S2 of the Supplementary material available on Dryad shows that the most significant linkages under our model are between closely functionally related genes. Our results confirm the strong relationship between evolutionary similarity and functional similarity between genes.

**Figure 6.**
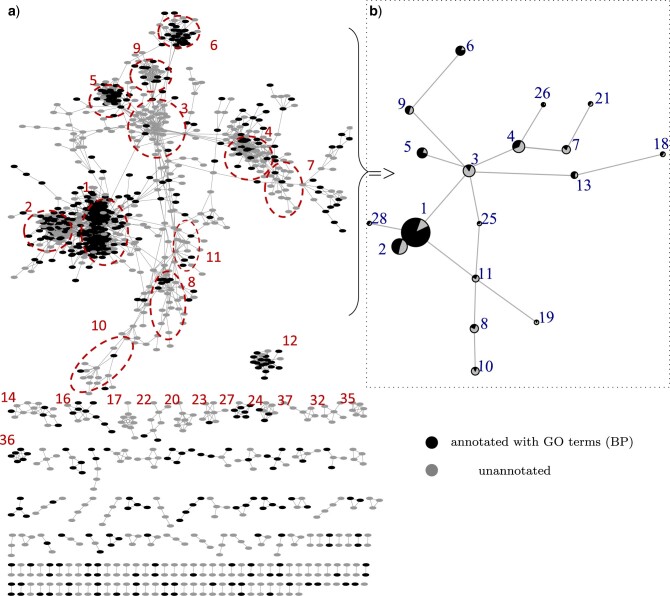
Visualization of the gene network: a) The gene network obtained from the full pairwise comparisons and labeled with the MCL clustering results. Black vertices indicate the genes annotated with GO (BP) terms and gray vertices denote unannotated genes. b) A detailed structure inside the largest component in (a). Each pie chart denotes the percentage of the annotated genes within each cluster. Only the clusters of size $> 5$ are labeled for a clean visualization.

To obtain the clusters of genes with highly correlated evolution, we firstly applied a strict threshold (coefficient of interaction $\beta_{12} > 0.75$ and Z score $>7.5$) on the linkages to obtain a gene network which consists of 1401 vertices and 19,391 highly significant (*P*-value$<6.37 \times 10^{-14}$) edges (Fig. 6a). We further applied Markov clustering with inflation parameter 1.5 on the network to provide a guidance for labeling the genes into clusters in the largest component (Fig. 6b). We reported the GO enrichment analysis for all the clusters of size at least five in Table S2 of the Supplementary material available on Dryad.

We do not expect profile similarity and clustering to align perfectly with participation in a common biological process, especially when biological processes are annotated at very low levels of specificity (e.g., “transmembrane transport”). Nonetheless, we expect that many genes with common functions (such as transmembrane transport, transcription, and carbohydrate metabolic process) will show similar distributions across genomes, reflecting processes such as hitchhiking on frequently transferred mobile elements and coincidental loss of genes that collectively confer no selective benefit. The flagellum cluster (Cluster 5) and amino-acid biosynthetic cluster (Cluster 6) were also discovered and examined in our previous study using Pagel’s correlation method applied on a reduced data set (a 74-tip subtree). It was only possible to analyze a reduced data set because of the computational cost of Pagel’s method, and a phylogenetic analysis was also conducted to find potential evidence for LGTs (Liu et al. 2018). In this study, by applying our method to the full data set (659 species), we discovered another candidate group of flagellar genes (Cluster 16) which are much less common (found in only 45 genomes) compared to the genes in Cluster 5 which are found in 396 genomes (Table S2 of the Supplementary material available on Dryad).

The intrinsic rates inferred by CCM were consistent with distribution patterns of genes in phylogenetic profiles. For example, the pattern in Cluster 4 appears to be more consistent with Darwin’s scenario, which is consistent with its relatively low intrinsic rate (Fig. S3 of the Supplementary material available on Dryad). Clusters 29 and 33 have the largest estimated intrinsic rates, and both show patchy distributions in the same very shallow clade in the tree. This rapid gain and loss over a relatively short span in the tree is a possible cause of the high rates. Cluster 36 (profiles in Fig. S4b of the Supplementary material available on Dryad) and Cluster 55 have the largest estimated interaction coefficients ($\beta$), and they both show strong functional associations according to their GO annotations as well. More detailed information about clusters can be found in Table S2 of the Supplementary material available on Dryad. To complete the analysis, we also provided a list of GO predictions on 823 unannotated genes based on most interacting genes that have known GOs, and the results are summarized in Table S3 of the Supplementary material available on Dryad.

#### Examples of inferred evolutionary relationships

The simulation results have shown that the pairwise comparisons could not detect the conditionally independent linkages, so that using all-versus-all pairwise comparisons tends to produce densely connected networks. For example, the five genes in Cluster 49 (Fig. S4a of the Supplementary material available on Dryad) are all related to iron–sulfur (Fe–S) assembly (three are annotated with “iron–sulfur cluster assembly,” one is annotated with “cysteine metabolic process” and one has no GO annotation but has the protein name “FeS assembly ATPase SufC”). The pairwise comparisons suggest that the linkages between all five genes are extremely strong (largest *P*-value $ 3.13 \times 10^{-9}$), which would lead to a fully connected network. However, by modeling these five genes as a community, 4 out of 10 total linkages can be removed as conditionally independent linkages (*P*-value $> 0.05$).

In other cases, the pairwise interactions are still significant even when we account for conditional dependence. As an example, Cluster 36 consists of six genes which are all annotated with GO term “alginic acid biosynthetic process.” The pairwise comparisons show that all links between the six genes are highly significant (largest *P*-value $6.43 \times 10^{-12}$). By modeling these six genes simultaneously as a community, only 3 out of 15 total linkages have a *P*-value $> 0.05$ as shown in Figure S4b of the Supplementary material available on Dryad.

**Figure 7.**
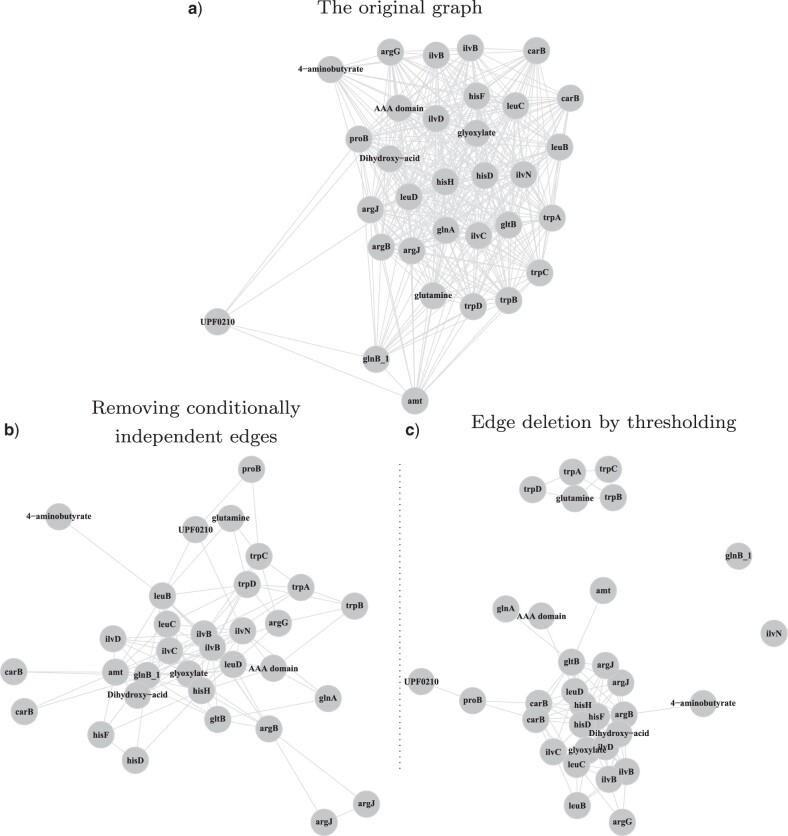
Network analysis of the amino-acid gene cluster. a) The original network (Cluster 6) consists of 32 vertices and 381 highly significant (*P*-value $<6.37\times 10 ^{-14}$) edges based on the all-versus-all pairwise comparisons (b) Application of the CCM on every triplet from network (a) followed by removal of the conditionally independent edges (*P*-value $>0.001$ and interaction coefficient $\beta$$<0.5$). The resulting network consists of 32 vertices and 109 edges. c) Direct deletion of edges from (a) by thresholding to retain the same number of edges as in (b). The cluster is disconnected into two components and two singletons. The force-directed layout algorithm is used for the network visualization.

Because the size of the transition matrix, and therefore the computational cost of our method, increases exponentially with the number of genes, it is infeasible to apply our method to large groups of genes. For large clusters, we get around this issue by applying our method to smaller cliques within the network, and using this to detect linkages that are conditionally independent. This is different from directly removing linkages by thresholding, as it aims to only remove the “redundant” linkages conditioning on other genes’ presences to reveal the refined structure rather than to break the cluster into smaller groups. For example, we started from the original network of Cluster 6 which consists of 32 amino-acid related genes and 381 highly significant linkages (*P*-value$<6.37\times 10 ^{-14}$) obtained from all-versus-all pairwise comparisons (Fig. 7a). Then we applied CCM over all the triplets within this network and some strong linkages became weakly significant due to the presence of the third gene. We removed 272 such edges (*P*-value $>0.001$ and interaction coefficient ($\beta$) $<0.5$) and obtained the refined network (Fig. 7b). For comparison, we directly deleted the same number of edges from the original graph by increasing the threshold (Fig. 7c). This results in a very different network structure consisting of multiple densely connected components, rather than a more sparsely connected network obtained using our method.

### Analysis of Mitochondrial Respiratory Complex I

**Figure 8.**
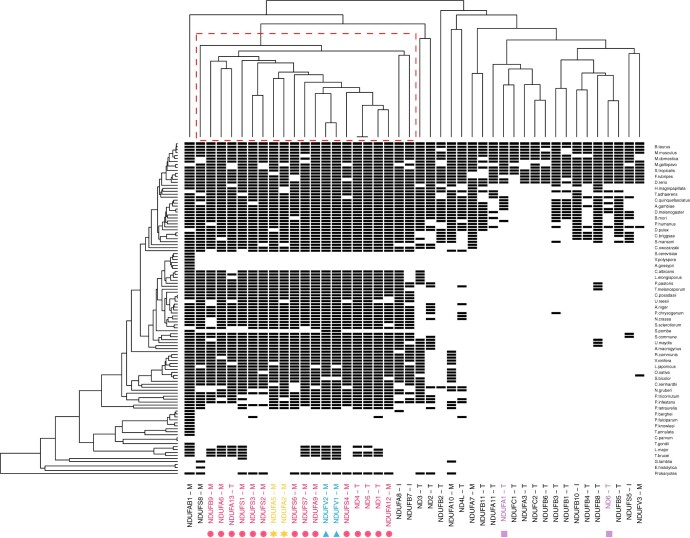
Clustering of mitochondrial respiratory complex I genes: the heatmap shows the phylogenetic profiles of 44 genes where black bars indicate presence. The column labels give the information of subunits, names - location (M, Matrix; T, Transmembrane; I, Intermembrane). The symbols below the gene names indicate the four components inferred from CLIME and those without symbols below indicate singletons. The dendrogram on the left indicates the eukaryotic tree and the names of species are given on the right as the row labels; the dendrogram above shows the hierarchical structure constructed with the estimated pairwise interactions by CCM.

Eukaryotic genes are less susceptible to LGT (Keeling and Palmer 2008; Sibbald et al. 2020), and we may therefore expect significant differences in the performance of CCM between prokaryotic and eukaryotic data. To evaluate the performance of CCM on eukaryotic data, we applied our CCM method on a well-studied protein complex which consists of a total of 44 human genes encoding Mitochondrial respiratory complex I (Balsa et al. 2012; Li et al. 2014; Guo et al. 2017). The data sets we used are published phylogenetic profiles and a species tree consisting of 138 diverse eukaryotes and a prokaryote outgroup (Bick et al. 2012; Li et al. 2014). We performed an all-versus-all comparison using CCM to infer the interactions among 44 genes and illustrate the detailed relationships within the complex with the average linkage hierarchical dendrogram as shown in Figure 8. We also compared our results with CLIME, an approach to infer evolutionary modules specifically for eukaryotic species which assumes that each gene must only have one single gain event in evolution followed by zero or more loss events. CLIME groups 20 of the 44 genes into four evolutionary modules (ECMs) with the remainder as singletons with no assigned group as shown in Figure 8 (the results of CLIME are available at https://gene-clime.org/). Comparing our clustering results to the detailed structure of complex I reported by Guo et al. (2017), we find a a single cluster of size 21 encompassing 15 genes that all localize to the matrix arm of CI including all 7 core subunits (NDUFV1, NDUFV2, NDUFS1, NDUFS2, NDUFS3, NDUFS7, and NDUFS8). The other main cluster includes 20 subunits, 15 of which localize on the membrane arm. We also analyzed the estimated evolutionary rates and find that the loss rates are significantly (*P*-value $< 2.2 \times 10^{-16}$) larger than the gain rates (Fig. S5 of the Supplementary material available on Dryad), which supports the idea that eukaryotic genes are much less mobile than prokaryotic genes. To further study the structure of complex I, we first obtained a network consisting of 462 significant (*P*-value$<0.05$) links that were inferred by full pairwise comparisons using CCM. After pruning the network by removing the conditionally independent links (*P*-value $>0.05$) detected from all triplets, we obtained a more sparse network consisting of 101 linkages (Fig. S6 of the Supplementary material available on Dryad). We can observe two loosely connected components in this network: one is mainly composed of more densely linked subunits on the matrix arm with higher estimated values for the coefficients of interaction, while the other component is mainly composed of the subunits on the membrane arm. This network representation of the gene-interaction map shows more comprehensive information about the gene evolutionary cohesiveness than the pure clustering results in Figure 8.

## Discussion

Identifying associations among traits is an important tool to generate hypotheses about linkages between phenotypic, ecological, and genetic attributes. Often these associations need to be further analyzed or tested experimentally to demonstrate whether they have arisen due to selection or other factors (Peiman and Robinson 2017). Phylogenetic profiles are a specialized type of trait representation that have been used for over 20 years as a tool to explore and compare genomes; while they can be treated in a similar fashion to other types of traits, the sequences, genetic linkage information, and functional annotations associated with genes in a profile can be used to shed more light on evolutionary hypotheses. Many studies suggest that phylogenetic relationships among source genomes should be taken into account (Pagel 1994; Cokus et al. 2007; Cohen et al. 2013; Liu et al. 2018). Our previous work demonstrated the utility of Pagel’s model (Pagel 1994) in identifying sets of genes with correlated evolutionary trajectories; however, this approach was computationally expensive and could not infer the direction of the relationship. In this study, we proposed a new coevolution model, CCM, to detect the genes with correlated evolutionary histories based on phylogenetic profiles. CCM was able to identify correlated genes as well as the direction of the relationship (e.g., Fig. S4a of the Supplementary material available on Dryad) and ran five times faster than Pagel’s method when tested on phylogenetic trees with 500 tips. The number of pairwise comparisons increases quadratically with the number of genes to be considered, but the independence of each comparison allows calculations to proceed in parallel. Heuristic methods can be used to quickly subdivide genes into large clusters that can then be refined using the CCM. Our model also has the ability to analyze the evolutionary relationships among sets of genes of size greater than 2. Examining sets of size $> 2$ can provide a more sparse gene network and greater insights of the complex relationships between genes.

Based on CCM, we also developed a simulation procedure that can generate a set of coevolved profiles with interactions along a given phylogenetic tree. The strength of the interactions during evolution is also adjustable. A common way to evaluate comparative methods for detecting genes with correlated evolutionary histories is measuring the functional similarities based on gene annotations such as GO terms (Radivojac et al. 2013) and KEGG pathways (Jothi et al. 2007). However, such evaluation is subject to annotation completeness and the correlated patterns may not always reflect shared function as expressed by GO annotations. Our coevolving simulation procedure provides a way to generate benchmark data for evaluating the comparative methods.

In the simulation study, our method outperformed the nonphylogenetic method (Jaccard Index) and the tree-aware methods (Pagel’s correlation model, run-adjusted methods, and clade-adjusted methods) in detecting the significant links (Fig. 2d). We showed that our method can distinguish between Darwin’s scenario and the replicated co-occurrence scenario (Fig. 3). We also demonstrated that pairwise comparisons cannot detect conditionally independent links and further showed the performance of CCM in recovering the community structures (Fig. 4).

Finally, we applied our method to 3786 profiles across 659 genomes and the results showed a strong positive relationship between the evolutionary similarity and functional similarity (Fig. S3 of the Supplementary material available on Dryad). We also identified the gene clusters with enriched functions (Table S2 of the Supplementary material available on Dryad) that can be used to better understand the functional roles of gene groups and predicted 823 unannotated genes based on their most interacting genes with known GO annotations (Table S3 of the Supplementary material available on Dryad). We also demonstrated using CCM to refine the network obtained from the pairwise comparisons by removing conditionally independent linkages (Fig. 7). In addition to analyzing prokaryotic data, CCM has also been successfully applied to a eukaryotic data set of the well-studied Human Complex I and the recovered associations mapped well onto the structural associations that exist in the complex (Fig. 8, Fig. S6 of the Supplementary material available on Dryad). The results show that CCM as a general comparative model can also be applied to eukaryotic data. Although our method is specifically used to analyze the phylogenetic profiles in this study, we think it can have wide applications in other fields such as to study phenotypes of species (Goberna and Verdú 2016), ecological habitats (Fierer et al. 2012), and metagenomic profiling (Aagaard et al. 2012).

The uniqueness of the CCM lies in the careful modeling of each gene’s instantaneous gain and loss rates dependent on the current states of other genes. In addition to improving our ability to identify related genes, the CCM directly models the dependence between related genes in the evolutionary process. The same idea can possibly be generalized to phylogenetic models to jointly estimate the transition rate matrix of each site based on the current states of its neighbor sites or other related sites. The dependence between different genes, or different sites within a single gene, is an underexplored area in phylogeny and molecular evolution, with the majority of models assuming independence of sites. By developing better-fitting models that incorporate the dependence between different genes, we expect to gain insights into the mechanisms driving this dependence.

We also met a challenge in extending our method to directly model larger communities. The state space $\mathbf{S}$ will increase exponentially as we include more genes into the community. Currently, we have successfully tested our method on communities of sizes less than 10, but two problems will arise if we include more genes: the huge memory requirements to store the $Q$ matrix of dimension $2^n \times 2^n$ and the long computation time for eigendecomposition of $Q$. We have found that if we reorder the rows and columns of the transition matrix, there exists a recursive structure: the $Q$ matrix can be written as a block matrix of the form $Q = \begin{pmatrix} A & B \\ B & A \end{pmatrix}$, where $B$ is an antidiagonal matrix and $A$ has the same recursive structure as $Q$, $A = \begin{pmatrix} A' & B' \\ B' & A' \end{pmatrix}$ ($B'$ is still an antidiagonal matrix and $A'$ is a block matrix). We can solve the first problem by storing the $Q$ matrix as a sequence of small “blocks,” but we have not found existing mathematical methods to solve the eigendecomposition of block matrices with such recursive structures. Our future work will explore the possible solutions to decompose the $Q$ matrix more efficiently so that the CCM method is scalable.

## Software availability

The R package evolCCM was written in R v4.0.2 and is available on Github (https://github.com/beiko-lab/evolCCM).

## Supplementary material

Data available from the Dryad Digital Repository: https://doi.org/10.5061/dryad.p8cz8w9rd.
